# Meat protein alternatives: exotic species

**DOI:** 10.1093/af/vfad070

**Published:** 2023-12-19

**Authors:** Óscar López-Campos

**Affiliations:** Science and Technology Branch, Agriculture and Agri-Food Canada, Lacombe Research and Development Center, Lacombe, Canada

## Introduction

Meat is derived from animal muscle and a wide range of diverse animals are considered suitable for human consumption, albeit dependent on the cultural context. Although pork, poultry, beef, and lamb are the main meat categories nowadays, the list of potential alternatives is substantially broader (e.g., capybara, camel, rabbit, kangaroo, camel, water buffalo, and game meats). In the current cultural and political scenarios that are shaping the meat market, the production, and commercialization of meat from alternative species is expected to acquire a significant share of the market that is currently dominated by pork, poultry, beef, and lamb meats. Meat from some of these alternative species may be highly desired in some cultures but unthinkable in others due to cultural taboos or emotional reasons.

Important considerations related to how meat is obtained and processed from each species need to be addressed for the successful commercialization and consumption of these alternative meat sources.

The present issue of *Animal Frontiers* aims to shed light on the production, processing, and quality traits of meat obtained from other species as compared to traits of meat from conventional species. This special issue tackles different important aspects of meat protein options beyond the conventional options (pork, poultry, beef, and lamb) such as capybara, camel, game, rabbit, kangaroo, donkey, and water buffalo meats.

López-Arévalo et al. (2023) provide an overview of the advances, challenges, and possibilities for sustainable use of capybara (*Hydrochoerus hydrochaeris*) in Colombia. The authors conclude that capybara populations have the potential to be used sustainably in the flooded savannas of the Colombian Orinoquia. The authors also discuss the need for effective policies to monitor and control commercial processes and sustainable use of capybara including the participation of harvesters and consumers.

In this special issue, Suliman (2023) presents a perspective article reflecting on the importance of camel meat as a promising future source of high-quality protein. Camel meat has value that has long been ignored for largely unsubstantiated reasons. The author highlights the major current constraints that must be addressed in order to successfully commercialize camel meat as a source of high-quality protein.

Segura Plaza et al. (2023) provide a manuscript on Game Meat and High-Resolution Magic Angle Spinning Nuclear Magnetic Resonance Spectroscopy: a traditional foodstuff vs. a novel analysis technology. One main conclusion that the authors reach in this manuscript is that the inclusion of game meat could diversify meat markets, hence carcass merit and meat quality parameters must be standardized to meet consumer requirements. On the other hand, enhancing the consistency of game meat quality parameters requires the implementation of new technologies such as the high-resolution magic angle spinning nuclear magnetic resonance to assess and characterize game meats.

Failla et al. (2023) infer that alternative animal species, including rabbit, kangaroo, deer, buffalo, donkey, and likely others, produce meat with low *N*-glycolylneuraminic acid (Neu5Gc) and high *N*-acetylneuraminic acid (Neu5Ac) content. Hence, these alternative meats could prevent chronic inflammation while also providing essential nutrients for humans.

Insights in yak meat are being also featured in the present special issue. Zhang et al. (2023) provide a thorough article that details the nutritional aspects of yak meat compared with cattle and sheep as well as the environmental benefits and market potential of raising yak. Furthermore, this feature article tackles the main challenges in the yak large-scale production.

The present special issue concludes with two reviews by Huerta-Leidenz and Rodas-Gonzalez (2023) and Rodas-Gonzalez and Huerta-Leidenz (2023), which compare water buffaloes (*Bubalus bubalis*) and cattle (*Bos indicus* and *Bos taurus*) in terms of biological endpoints and meat quality, respectively. Despite much interest in the meat science field regarding similarities and differences between water buffalo and cattle, only a few studies have tackled this topic to date. The currently available data suggest that buffalo meat has satisfactory production traits, which are comparable to those of cattle. Furthermore, according to the literature reviewed, buffalo meat and beef are quite similar from a nutrient composition standpoint when produced under comparable conditions.

## About the Author

**Dr. Óscar López-Campos** is a Livestock Carcass Merit and Market Competitiveness research scientist at the Agriculture and Agri-Food Canada Lacombe Research & Development Center (AAFC-Lacombe RDC). He completed his undergraduate studies in Animal Science in 2002, then entered into graduate studies and received a PhD in Animal Production in 2007 at the University of León (Spain). He also worked in research at the Mountain Cattle Institute of the Spanish National Research Council, completed a 3-yr Natural Sciences and Engineering Research Council of Canada post-doctoral fellowship, and over 2-yr term as a research associate at the AAFC-Lacombe RDC. The major objectives of Dr. Óscar López-Campos’ Livestock Carcass Merit and Market Competitiveness program are focused on identifying measuring, prioritizing, and utilizing objective and subjective carcass merit and meat quality assessments and attributes of economic importance to promote development, adaptation, and competitiveness of the whole meat value-chain in Canada. Dr. López-Campos is also member of the editorial boards of Meat Science and Canadian Journal of Animal Science. **Corresponding author:**oscar.lopezcampos@agr.gc.ca
